# Multiple brain abscesses caused by *Nocardia asiatica* co-infection with Torque teno virus in an “immunocompetent” patient: a rare case report and literature review

**DOI:** 10.3389/fmed.2025.1661345

**Published:** 2025-11-12

**Authors:** Weiwei Huang, Xu Ran, Zuoxin Zhang, Lin Yang, Jinbo Yin, Shengqing Lv, Guolong Liu, Yuchun Pei

**Affiliations:** 1 Department of Clinical Laboratory, Xinqiao Hospital, Army Medical University (Third Military Medical University), Chongqing, China; 2 Department of Neurosurgery, Xinqiao Hospital, Army Medical University (Third Military Medical University), Chongqing, China

**Keywords:** multiple brain abscess, *Nocardia asiatica*, Torque teno virus, immunocompetent, mNGS

## Abstract

Brain abscess is a suppurative infection of brain tissue caused by one or more pathogens under specific susceptible conditions and is associated with a high clinical fatality rate. Beyond surgical intervention, the identification of pathogens is key to clinical antimicrobial therapy, yet this remains a challenge. *Nocardia* is a ubiquitous bacterium that typically manifests as an opportunistic infection, primarily affecting immunocompromised individuals. Pulmonary involvement, characterized by suppurative inflammation, commonly occurs following inhalation, with subsequent hematogenous dissemination potentially leading to widespread infection. To our knowledge, central nervous system (CNS) infection by *Nocardia asiatica* (*N. asiatica*) resulting in brain abscess has hitherto rarely been reported. We present a rare case of multiple brain abscesses caused by *N. asiatica* co-infection with Torque teno virus (TTV) in an immunocompetent patient with suspected multiple organ involvement. The patient was admitted to our hospital, presenting with a headache, and imaging revealed brain abscess-like lesions. A robot-assisted stereotactic puncture and drainage were used for abscess removal. *N. asiatica* and TTV were identified by metagenomic next-generation sequencing (mNGS) of the brain abscess aspirate, with *N. asiatica* subsequently confirmed by mass spectrometry of the cultured organism. A disseminated *Nocardia* infection was suspected based on the patient’s skin trauma history, pulmonary inflammatory changes, and imaging findings (liver cysts, subcutaneous nodules). However, etiological confirmation was not obtained prior to his death. While this is not the first reported instance of *Nocardia* and TTV co-infection in brain abscesses, our case is notable for its occurrence in an immunocompetent patient. This report highlights the significance and value of TTV in the context of brain abscesses and warrants a re-evaluation of *Nocardia* and TTV co-infection. Given that the diagnosis of intracranial infection depends on the detection of pathogens, we advocate for the routine and early implementation of mNGS testing in patients with brain abscesses. Moreover, systemic nutritional support and immunomodulatory therapies should be considered in the early stage of treatment for complex cases. Earlier diagnosis and treatment in this case might have altered the patient’s outcome.

## Introduction

Brain abscess is a life-threatening disease with high fatality rates (1, 2). It usually arises from inflammation caused by bacteria, mycobacteria, fungi, or parasites (3). The pathogenesis of brain abscess is related to predisposing factors, such as contiguous infections, head trauma, neurosurgery, and hematogenous spread in patients with systemic infection or immunocompromise (3). Clinical manifestations of brain abscess are often nonspecific, including headache, fever, neurological deficits, nausea, vomiting, and altered consciousness, which can result in diagnostic delay (4). *Nocardia*, as a rare intracranial pathogen, has received relatively little attention.

*Nocardia* is a strictly aerobic Gram-positive mycobacterium that may exhibit variable or weak staining with standard Gram stain and is weakly positive with modified acid-fast staining (5, 6). As a conditional pathogen, *Nocardia* is widely distributed in nature and typically causes exogenous infections in immunocompromised hosts (5). Lung, skin, and soft tissue are the most attacked areas for this bacterium. It can also be introduced into the central nervous system (CNS), leading to brain abscesses mostly through hematogenous spread (7). *Nocardia* infections account for only 2% of all reported bacterial brain abscesses (7). About 41% of brain abscesses caused by *Nocardia* are multiple, with a mortality rate over 60%, significantly higher than the mortality rate of single brain abscesses (33%) and brain abscesses caused by other bacteria (<10%) (8). *Nocardia cyriacigeorgica* (*N. cyriacigeorgica*) and *Nocardia asteroids* (*N. asteroides*) are the most commonly isolated species (5). Few cases of *Nocardia asiatica* (*N. asiatica*) causing brain abscesses have hitherto been reported. The insidious nature of cerebral nocardiosis often leads to misdiagnosis and delayed treatment, making its diagnosis and treatment highly challenging.

Torque teno virus (TTV), a human-associated DNA virus reported in 1997, was initially believed to be linked to post-transfusion hepatitis (9). It is now recognized as a ubiquitous agent, detectable in the plasma of over 80% of the population, including healthy individuals (10). Although TTV has been detected in a variety of human samples, there is no clear evidence of its pathogenicity in humans (11). At present, TTV is generally regarded as a commensal virus given its long persistence in the body without clinical symptoms. However, in hematopoietic stem cell transplantation (12) and solid organ transplantation studies (13), TTV has great potential as an immune biomarker and can be used to monitor the immunosuppressive intensity of transplant recipients. Although some studies have found TTV in the cerebrospinal fluid (CSF) of patients with neurological diseases (14, 15), its role among CNS diseases still requires extensive research.

Brain abscesses commonly present as single or multiple lesions, mostly caused by pyogenic bacteria, mycobacteria, fungi, and parasites. However, the phenomenon of bacterial and viral co-infection in this context is not well understood, and its recognition may have different implications. Here, we report a case of multiple brain abscesses caused by co-infection with *N. asiatica* and TTV in an immunocompetent patient with suspected disseminated nocardiosis involving the lungs and liver. This rare case provides new insights into *N. asiatica* and TTV co-infection and may contribute to a more comprehensive understanding of the diagnosis and treatment of multiple brain abscesses. Furthermore, the relationship between viruses and bacteria remains largely unknown. In this case, TTV may have contributed to a decline in the patient’s immunity, thereby aggravating the condition. This case prompts a renewed consideration of the role of TTV in brain abscesses.

## Case presentation

A 52-year-old male presented to our hospital in April 2023, complaining of continuous headaches for more than 10 days without any predisposing factors, with left-sided gait disturbance for 4 days, accompanied by nausea. Prior treatment at a local hospital yielded no significant improvement. His medical history was unremarkable for hypertension, diabetes mellitus, coronary artery disease, or stroke.

Two months prior to admission, the patient was admitted to another hospital for pulmonary masses. Contrast-enhanced computed tomography (CT) of the chest showed a 38 mm × 26 mm lobulated mass in the anterior segment of the upper lobe of the right lung, with spiculated margins and mildly heterogeneous enhancement with internal punctate calcification. Besides, the upper lobe and middle lobe of the right lung exhibited scattered ground-glass opacity and linear shadows. Solid nodules were observed in the apicoposterior segment (8.5 mm × 6.7 mm) of the left superior lobe and the right oblique fissure (7.2 mm × 5.7 mm) of the left lung, without significant enhancement. No enlarged lymph nodes were found in the hilar and mediastinal regions. Upper-abdominal CT imaging revealed a circular hypodense lesion about 18 mm × 9 mm in size in the S6 segment of the liver, along with another high-density nodular shadow in the right lobe of the liver. Head CT demonstrated an empty sella, with no other significant intracranial abnormalities. Based on the suspicion of a tumor in the upper lobe of the right lung, thoracoscopic pulmonary lobectomy and pleural adhesion release were performed. Histopathological examination reported extensive infiltration of acute and chronic inflammatory cells in the lung tissue, focal fibrous tissue hyperplasia, accompanied by atypical alveolar epithelial hyperplasia.

Upon admission to the neurosurgery department of our hospital, a physical examination showed dysarthria, slow response, and limited cooperation. The neurological assessment demonstrated decreased muscle tone in the left upper and lower extremities with grade 4 muscle strength and a hemiplegic gait. The relevant laboratory test results are presented in Table 1.

**Table 1:** Laboratory results on admission.

Laboratory test	Reference range	Measurements
Routine bloodwork		
WBC (×10^9^/L)	3.5–9.5	14.6
Monocyte (×10^9^/L)	0.1–0.6	1.21
Neutrophils (%)	40–75	77.3%
Lymphocytes (%)	20–50	14.1%
Inflammatory index		
CRP (mg/L)	0–8	5.34
ESR (mm/h)	0–15	10
IL-6 (pg/mL)	0–0.34	9.7
TNF-α (pg/mL)	0–8.1	9.3
Tumor marker		
CEA (ng/mL)	0–5	0.69
CSF examinations		
WBC (×10^6^/L)	0–8	49
Protein (g/L)	0–0.45	1.14
Glucose (mmol/L)	2.5–4.5	4.96
HIV testing		
HIV antibody	Negative	Negative
HIV-P24 antigen	Negative	Negative
Microbial cultures		
Boold	No growth	No growth
CSF	No growth	No growth

WBC, white blood cell; CRP, C-reactive protein; ESR, the erythrocyte sedimentation rate; IL-6, interleukin 6; TNF-α, tumor necrosis factor-α; CEA, carcino-embryonic antigen; CSF, cerebrospinal fluid; HIV, Human Immunodeficiency Virus; mNGS, metagenomic next-generation sequencing.

Contrast-enhanced CT of the chest revealed a funiform high-density shadow in the right lung after the operation. The hilar shadow of the right lung appeared enlarged, with unclear borders, and no definitive abnormal enhancement was observed.

**Figure 1:**
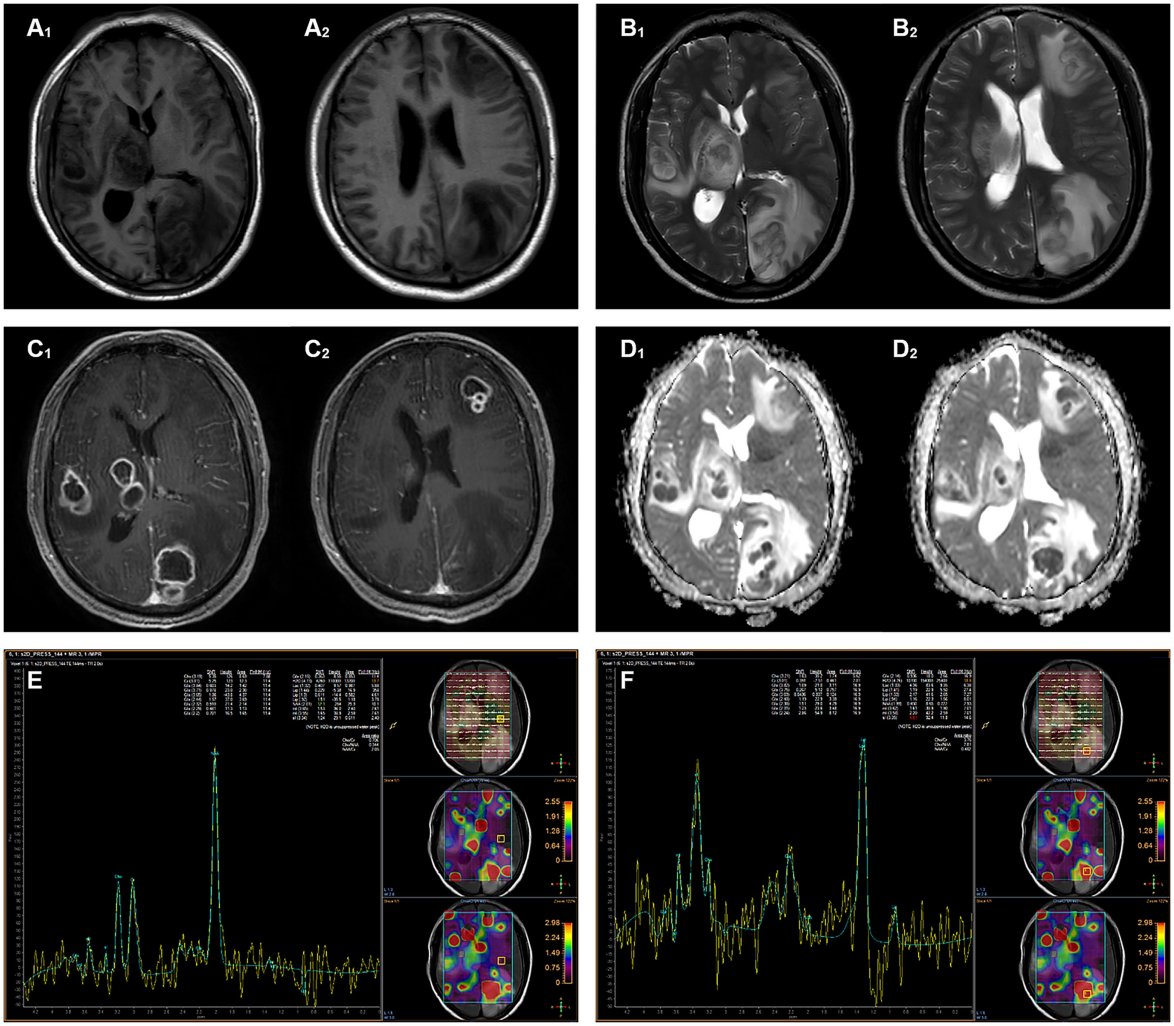
Magnetic resonance imaging shows multiple intracranial lesions. **(A₁,A₂)** The lesion areas demonstrate hypointense and isointense on T1WI. **(B₁,B₂)** The lesion areas demonstrate hyperintense and isointense on T2WI. **(C₁,C₂)** The signal intensity of the ring wall was increased after enhancement. **(D₁,D₂)** DWI hyperintensity with ADC hypointensity, the lesion shows true diffusion restriction. **(E)** MRS shows normal metabolite peaks within the normal area. **(F)** MRS of the lesion demonstrates a pathological spectrum, suggestive of abscess.

Head magnetic resonance imaging (MRI) indicated multiple lesions in the left frontal lobe, parietal occipital lobe, temporal lobe, right temporal lobe, thalamus, and brain stem with low signal intensity on T1-weighted imaging, high signals on T2-weighted, and hyperintensity on T2-weighted fluid-attenuated inversion recovery (FLAIR) sequences. Diffusion-weighted imaging (DWI) revealed distinct hyperintense regions centrally within the lesions, corresponding to hypointense regions on apparent diffusion coefficient (ADC) mapping. Patchy edema was observed around the lesions, with the largest lesion located in the left parieto-occipital lobe, measuring about 35 mm × 23 mm. Mild localized compression of the third ventricle and left lateral ventricle was noted, along with a partial midline shift to the left. The peripheral enhancement of multiple lesions was observed after contrast administration. Magnetic resonance spectrum (MRS) showed spectral alterations consistent with multiple intracranial lesions (Figure 1).

Owing to the patient’s limited cooperation, positron emission tomography-computed tomography (PET-CT) examination could not be adequately performed. The results showed multiple hypodense shadows in both cerebral hemispheres, with ring-enhancing lesions in the right thalamic region and the left occipital lobe. The largest size of these measured approximately 16 mm × 12.5 mm and exhibited low density in the center with peripheral high density. The increased F-fluorodeoxyglucose (FDG) metabolism in these lesions indicated infectious lesions. The lymph nodes of the right hilar region and the mediastinum at the 7th thoracic vertebral level showed increased FDG metabolism, suggestive of reactive hyperplasia. The right lobe of the liver exhibited a low-density lesion, measuring 16.1 mm × 16.8 mm, and showed no abnormal increase in FDG uptake, indicative of a cyst, which appeared larger than on previous imaging 2 months ago. The left lobe of the liver exhibited a high-density punctate lesion. Increased FDG uptake of the left adrenal gland was attributed to reactive hyperplasia or physiological uptake. Bundled ingestion of radioactivity was noted within the intestinal tract. Multiple high-density nodular shadows were observed subcutaneously on the right hip.

Based on the results above, the patient received a diagnosis of multiple brain abscesses and was initiated on empiric antibiotic treatment with imipenem and amikacin. Drainage of multiple intracranial abscesses by robot-assisted stereotactic under general anesthesia was performed. Twelve hours later, the patient’s condition worsened. Emergency head CT examination revealed multiple low-density intracranial shadows, high-density images in the right temporal lobe, some cisterna, and sulci, with multiple intracranial gas foci. These findings were suggestive of cerebral hernia caused by swelling of intracranial brain tissue, prompting emergency lateral ventricle drainage performed at the bedside. The antibiotic regimen was then adjusted to meropenem and vancomycin for continued empirical anti-infective treatment.

Ten milliliters of pale-yellow pus with no distinct odor were aspirated during the surgical procedure. Hematoxylin and eosin (HE) staining of the pus specimen showed focal brain necrosis and extensive lymphocyte infiltration, along with glial cell proliferation with bleeding and edema.

The pus specimen was subjected to commercial high-throughput metagenomic next-generation sequencing (mNGS) utilizing the PMseq service (BGI Genomics, Shenzhen, China). Total DNA was extracted from the specimen using the TIANamp Micro DNA Kit (DP316, TIANGEN BIOTECH, Beijing, China). Sequencing libraries were constructed through fragmentation, end repair, adapter ligation, and PCR amplification. The libraries were sequenced on the MGISEQ-2000 platform in a 50 bp single-end mode. Following the computational removal of low-quality reads and human host sequences (aligned to the hg19 reference genome using Burrows-Wheeler Alignment), the high-quality data were aligned to the Pathogen Metagenomic Database (PMDB) for taxonomic classification. On the following day, the mNGS results identified 1,863 *N. asiatica* gene sequences, representing a relative abundance of 79.84%, and 5 TTV gene sequences with a relative abundance of 100%. The raw sequence data were deposited into the NCBI SRA under the accession number PRJNA1330259.

**Figure 2:**
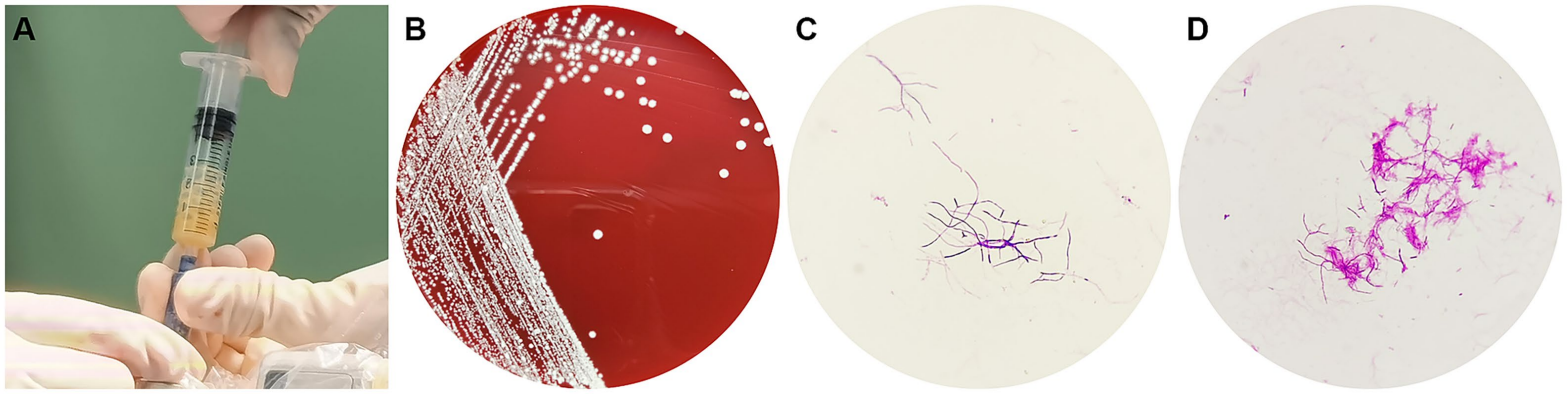
**(A)** A pale yellow pus was aspirated during the operation, **(B)** White bacterial colonies grown on a blood agar plate, **(C)** Gram-positive branching filaments observed in a cultured colony, **(D)** Weakly positive modified acid-fast staining of a cultured colony.

After 3 days of aerobic culture of the abscess secretions, white villous colonies were observed on blood agar, Gram staining showed branching filamentous bacteria, and modified acid-fast staining was weakly positive (Figure 2). *N. asiatica* was further identified by matrix-assisted laser desorption ionization-time-of-flight mass spectrometry (MALDI-TOF MS). The anti-infective treatment regimen was adjusted to include compound trimethoprim/sulfamethoxazole (TMP-SMX) and acyclovir.

**Figure 3:**
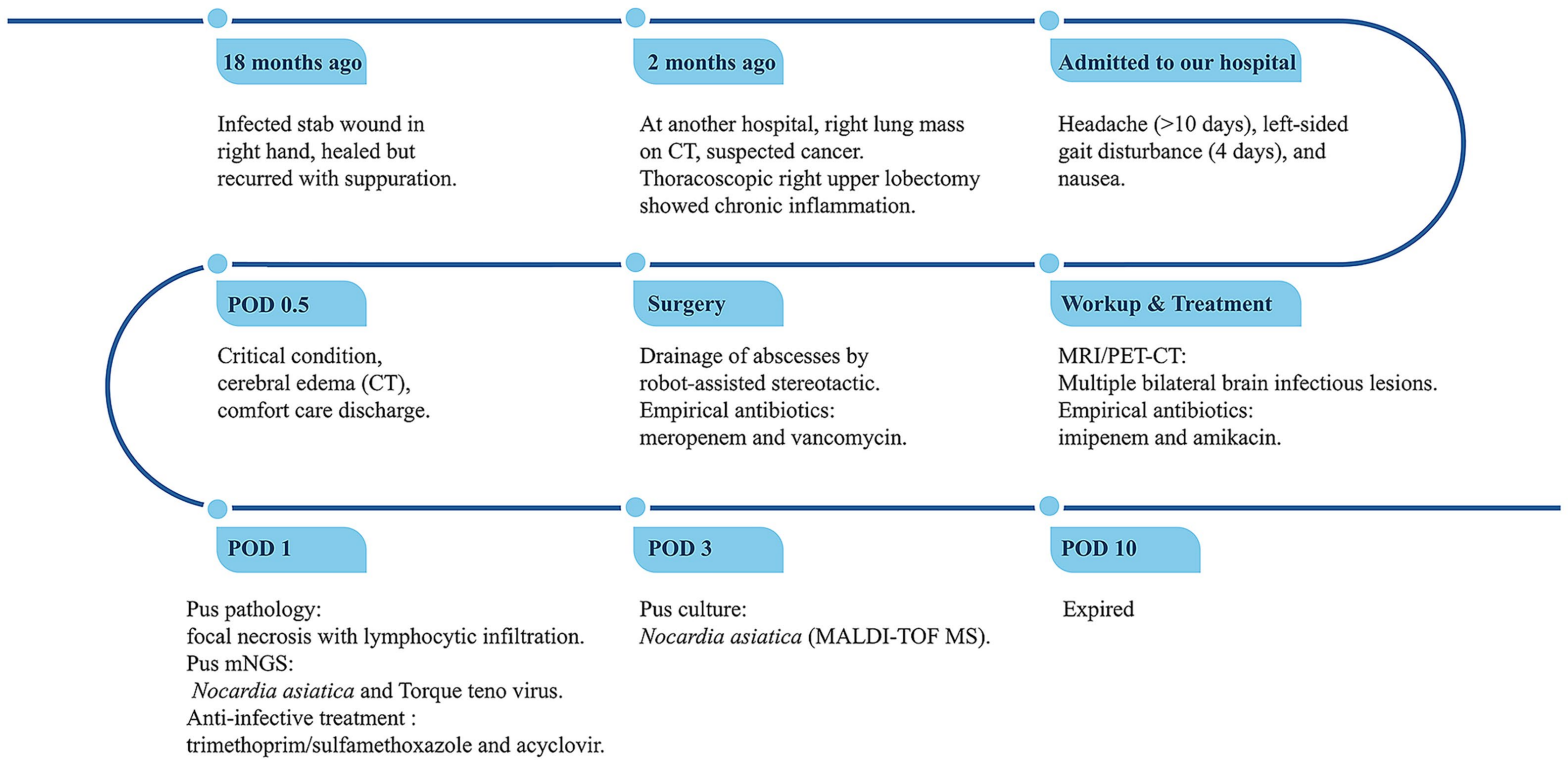
Timeline of disease progression and treatment. POD, Postoperative Day; mNGS, metagenomic next-generation sequencing.

Unfortunately, the patient remained critically ill after the operation, and despite resuscitative efforts, he died 10 days later. The clinical timeline is summarized in Figure 3.

## Discussion

Brain abscess is a serious, life-threatening infectious disease of the CNS. In individuals with compromised immunity or the presence of a potential source of infection, pathogens can invade the brain tissue in different ways, leading to the formation of a necrotic purulent cavity, and inducing peripheral inflammation and extensive cerebral edema. Given its high mortality and disability rates, identifying the causative pathogen and early treatment are key to improving the prognosis of the disease. Current evidence suggests that 23% of brain abscesses involve co-infection with multiple microorganisms (16), and the increasing identification of rare pathogens underscores the significant areas of knowledge regarding brain abscesses that remain to be explored.

Nocardiosis is an acute or chronic suppurative or granulomatous disease caused by *Nocardia* bacteria, which is commonly observed in immunocompromised hosts, such as cancer patients, organ transplant recipients, and HIV-positive patients (17). Generally, HIV positivity indicates an immune deficiency, while HIV negativity indicates a normal immune status. Although *Nocardia* is considered an opportunistic pathogen, studies have shown that approximately one-third of cases have no underlying predisposition (18). In this case, the patient exhibited normal immune function with no risk factors for immunocompromise, consistent with Heer H. Mehta et al.’s observation that immune status is not the only determinant for infection with *Nocardia* (17). Our study is the first reported case of multiple brain abscesses caused by *N. asiatica* since 2010 in an immunocompetent individual without underlying disease (Table 2) (19–25).

**Table 2:** Literature review of brain abscess caused by *Nocardia asiatica*.

Year of diagnosis	Age/sex	Predisposing factors	Brain abscess	Other organs involved	Identification method	Treatment	Outcome
2012 (19)	49 M	Malignant thymoma, Myasthenia gravis	Multiple	Heart, anterior mediastinum	Not provided	LEV→IPM, AMK, TMP/SMX Pericardiocentesis, Debridement of the anterior mediastinum	Alive (5 months)
2017 (20)	51 M	SLE	Multiple	No	16S rRNA sequencing secA1sequencing	TMP/SMX, CRO No surgery	Expire (10 months)
2019 (21)	37 M	HIV	Multiple	Lung, skin, pancreas	16S rRNA sequencing	AMK, MEM → TMP/SMX Surgery unspecified	Alive (2 months)
2020 (22)	53 M	HIV	Isolated cerebellar brain abscess	No	MALDI-TOF MS; 16S rRNA sequencing	TMP/SMX Right suboccipital craniotomy with abscess drainage	Alive (3 months)
2021 (23)	61F	Left breast cancer resection, Diabetes mellitus	Signer	No	mNGS	LZD, MEM → LZD, TMP/SMX Excision of the cranial masses	Alive (1 year)
2023 (24)	63 M	Type 2 diabetes mellitus (T2DM), Adult-onset Still’s disease (AOSD)	Multiple	Skin and lung are unproven	16S rRNA sequencing	TZP, quinolone→TMP/SMX Stereotactic brain biopsy of the lesions	Expired
2023 (25)	27F	Atypical membranous nephropathy	Multiple	Lung	mNGS	MEM, TMP-SMX Craniotomy for the resection of lesions	Alive
Present case	52 M	Normal immune function	Multiple	Skin, lung, and liver are unproven	mNGS, MALDI-TOF MS	IPM, AMK → MEM, VA → TMP/SMX Puncture drainage of intracranial abscesses	Expired (10 days)

M, Male; F, Female; IPM, imipenem; AMK, amikacin; TMP/SMX, trimethoprim/sulfamethoxazole; CRO, ceftriaxone; MEM, meropenem; LZD, linezolid; TZP, piperacillin/tazobactam; VA, vancomycin.

*Nocardia* is widely found in air, soil, water, decaying vegetation, and animal excrement (26, 27). The mycelium fragments formed by *Nocardia* can enter the lung through the respiratory tract (27) or through direct contact with damaged skin to cause exogenous infection and then spread to the CNS through the bloodstream (28). Typically, one or more brain abscesses form when the bacteria invade the brain parenchyma (28, 29). The clinical symptoms in patients are generally insidious and variable, mainly manifesting as headache, nausea, vomiting, confusion, and increased intracranial pressure. Some patients may experience seizures (29). Moreover, cases of multiple brain abscesses caused by *N. asiatica* have rarely been reported. *N. asiatica* infections involving multiple organs by hematogenous dissemination are even more unusual (19).

Cranial imaging, including CT or MRI, can help to identify brain abscesses, which typically manifest as ring-enhancing lesions. Functional MRI enables us to distinguish from other annular enhancement lesions such as tumors, cystic foci, or necrotic foci (3, 30, 31). In the present case, the patient was hospitalized due to symptoms such as headache, nausea, and left walking. Both PET-CT and MRI showed multiple focal images with enhanced annular edges. In addition, the DWI of lesions showed significant diffusion restriction with high signal intensity, and corresponding regions showed low signal intensity on ADC. Based on these findings, the patient was diagnosed with multiple intracranial abscesses.

Given the diagnostic challenges posed by the often atypical clinical manifestations of *Nocardia* infection (32), the pathogen must be detected to establish a definitive diagnosis. In recent years, mNGS, as a new molecular biological method, has a comprehensive and direct advantage over culture, antigen detection, and PCR methods for the clinically accurate diagnosis of complex infectious diseases. It can quickly detect all nucleic acid information from clinical samples and identify various pathogens, including viruses, bacteria, fungi, or parasites, down to the species level without targeting (33). While culture remains the gold standard, *Nocardia* typically requires 3 to 7 days for growth and necessitates high-quality pus specimens, making its isolation by traditional culture methods challenging. Considering the patient’s clinical status and the need for etiological confirmation, urgent surgical intervention was required. Intraoperative pus extraction was initially identified as *N. asiatica* and TTV by mNGS within a day, while the cultured colonies were subsequently confirmed by MALDI-TOF MS 2 days after mNGS results were obtained.

Recently, surgical drainage has emerged as one of the crucial methods for the diagnosis and treatment of *Nocardia* infection in the CNS in some cases (3, 30). Advances in neurosurgical techniques have facilitated stereotactic puncture of abscesses at least 1 cm in diameter, regardless of location (3). Multiple abscesses require extensive surgical treatment (8). In addition, antibiotic therapy remains the main form of treatment. In this case, the patient presented with bilateral multiple brain abscesses requiring surgical intervention and empirical antibiotic therapy. Preoperative examination indicated that the patient had a high risk of intracranial infection and empiric treatment with imipenem and amikacin. Meropenem and vancomycin were given postoperatively. When *N. asiatica* and TTV were identified, targeted therapy was implemented with oral TMP-SMX and acyclovir.

Upon further inquiry into the patient’s medical history, it was revealed that he sustained skin trauma in the latter half of 2021. Specifically, a thorn penetrated deeply into his right ring finger while he was in a mountainous area. The thorn was not removed until the wound had become infected, and recurrent suppuration occurred even after the initial wound healing. Given the widespread distribution of *Nocardia* in the soil and environment, we hypothesize that the traumatic injury served as the portal of entry for the patient’s initial infection with *N. asiatica*. Studies have shown that primary cutaneous nocardiosis often invades immunocompetent hosts and is not restricted to specific *Nocardia* strains (29). Usually, the bacteria may be present for more than half a year before the onset of clinical symptoms. Cutaneous *Nocardia* infection can not only form local abscesses but also deeply invade the lymphatic system and form subcutaneous nodules (29). Based on the above findings, it can be inferred that *N. asiatica* was inoculated through the compromised skin barrier, proliferated to cause subcutaneous lesions, and subsequently disseminated to the CNS via hematogenous spread. Further evaluation is warranted to determine the presence of other disseminated lesions.

In our case, approximately 18 months elapsed between the patient’s initial finger infection and the onset of neurological symptoms leading to the diagnosis of *N. asiatica*, which could be explained by the insidious nature of *Nocardia* infection. Upon initial evaluation at another hospital, CT imaging revealed multiple pulmonary nodules and hepatic cysts. Due to irregular pulmonary enhancement on CT, the patient was suspected to have lung cancer and subsequently underwent surgical intervention. However, no etiological investigation was conducted, and no antibiotic therapy was initiated despite pathological findings indicating inflammatory changes. Indeed, had the underlying cause been identified at that time, the patient’s treatment course and outcome would likely have been significantly different. Upon presentation to our hospital, imaging demonstrated multiple intracranial abscesses, hepatic nodules with cyst enlargement, and subcutaneous nodules in the gluteal region. Although the patient had no prior significant medical history, the pneumonectomy performed 2 months earlier, coupled with untreated and uncontrolled infection, likely contributed to persistent inflammation, ultimately exacerbating the disseminated infection and culminating in parenchymal invasion of the cerebral tissue. This progression suggests that compromised immunity may have predisposed the patient to disseminated nocardiosis or exacerbated its severity. The clinical and radiological findings are consistent with disseminated nocardiosis affecting the skin, lungs, liver, and brain. However, this remained a presumptive diagnosis, as definitive etiological confirmation was not obtained. Before further diagnostic workup could be completed, the patient developed severe cerebral herniation postoperatively and underwent decompressive treatment before discontinuing care in the hospital. Tragically, the patient succumbed to the illness 10 days after discharge. Due to the family’s refusal to authorize an autopsy, we were unable to obtain microbial test results and pathological confirmation from the remaining tissues.

Meanwhile, besides *N. asiatica*, 100% TTV sequences were detected in the brain pus of the patient. At present, there is no direct evidence substantiating that TTV can cause clinical diseases. However, recent research has found that TTV is associated with co-infections of various diseases, such as multiple sclerosis (MS) and respiratory diseases. It has been reported that high levels of TTV viremia in chronic obstructive pulmonary disease patients lead to higher DNA damage (34). TTV-DNA was detected in serum samples of MS patients during the disease exacerbation stage (35). In addition, some recent studies have shown that TTV-DNA and TTV antibodies were detected in the CSF of encephalitis patients, suggesting that TTV may replicate in the CSF and participate in CNS pathology (36). Similarly, the detection of TTV or TTMV in the CSF of children with unexplained encephalitis indicates that these viruses may have played an infectious role (37). There have been reports of rapid clinical deterioration in patients with simultaneous detection of *Streptococcus intermedius* and TTV in CSF (38). The above evidence suggests that TTV may be involved in the progression and pathological damage of the disease, which prompts consideration in our case.

The detection of TTV in the brain abscess in this case is not an isolated finding. In 2024, Yuting Gu et al. simultaneously detected TTV and *Nocardia farcinica* in the pus of a female brain abscess patient using mNGS (39), indicating that the presence of TTV may not be coincidental. This might be due to several reasons. First, in the past, due to the limitations of traditional detection technologies, the clinical attention on brain abscesses primarily focused on bacteria, mycobacteria, fungi, or parasites; viruses in brain abscesses remain largely understudied. However, with the widespread application of mNGS, viruses can also be detected. In contrast, mNGS addresses these limitations by enabling the comprehensive detection of all pathogen sequences within a sample, including previously unknown viruses, thereby facilitating the discovery of a broader range of potential pathogens (33, 40). Additionally, TTV is a commensal virus in human blood and bodily fluids and persists for a long time without clinical manifestations. Therefore, autologous TTV can translocate to the CNS and then act as a conditional pathogen. Furthermore, the physical trauma from the lung resection surgery, combined with the undetected and uncontrolled *Nocardia* infection, may lead to a decline in the body’s immunity, providing an opportunity for exogenous or latent TTV to spread to the CNS. TTV may have gained access to the CNS by exploiting the disruption of the blood–brain barrier following severe craniocerebral trauma resulting from the *N. asiatica* brain abscesses, or through other less-understood mechanisms such as the lymphatic system or exosome-enriched vesicles.

Consequently, a renewed recognition of the significance and value of TTV in brain abscesses is warranted. Given the significant lymphocyte infiltration and glial cell proliferation observed in the pathology of brain abscesses, a slightly increased total WBC count in the CSF can suggest viral infection, although these findings are not specific. Moreover, the therapeutic effect of treating *Nocardia* alone was not satisfactory. The patient’s condition progressed rapidly. From the onset of neurological symptoms 2 months after the lung resection to the diagnosis of multiple intracranial abscesses, despite aggressive treatment strategies, the patient ultimately succumbed due to irreversible neurological damage caused by cerebral edema, leading to the failure of vital signs. These indicate that the coexistence of TTV and *Nocardia* may lead to more aggressive disease than isolated *Nocardia* infection.

Indeed, the presence of the virus could indicate a decline in the patient’s immunity and exacerbate the development of the disease. Therefore, TTV may not only be an indicator agent reflecting the actual immune status of the patients but also an essential factor leading to the progression and deterioration of the disease. We emphasize the importance of acknowledging the presence of TTV in disease states and propose that TTV can be utilized as a marker for evaluating immunosuppression in neurological diseases, extending its utility beyond the realm of transplantation.

A major limitation of this study is the absence of mNGS-based detection of TTV in the patient’s peripheral blood samples, which prevents the definitive establishment of the TTV source and its route of infection. Moreover, *in vitro* culture of TTV is challenging due to the absence of suitable cell lines, and the lack of relevant serological assays hinders the verification of specific antiviral immune responses. These limitations make it difficult to evaluate the potential pathogenic characteristics of TTV. So, further research is warranted to confirm these hypotheses. Ultimately, simultaneous consideration of the identified pathogens and the patient’s actual immune status could offer a more valuable approach to treatment, and neglecting these factors may have been a contributing factor to the patient’s death.

## Conclusion

Here, we report a rare case of multiple brain abscesses caused by *N. asiatica* co-infection with TTV, offering evidence to re-evaluate the role of viruses in brain abscesses. This case underscores the utility of mNGS in rapidly providing comprehensive etiological information when multiple lesions of unclear origin are present throughout the body. Therefore, we recommend mNGS testing of CSF as a routine diagnostic procedure for the early diagnosis of patients with brain abscesses, given its great reference value when abscess samples cannot be obtained in the early stages. In addition, emphasis should be placed on the role of TTV in brain abscesses, since it may lead to a decline in immunity and be involved in disease progression. This case prompts us to explore and improve the methods for detecting viruses, including RNA viruses. On the other hand, in patients with suspected immunosuppression, treatment strategies should include measures to enhance immunity, such as pharmacological interventions or the infusion of fresh plasma in addition to antibiotic treatment. Such a comprehensive approach may ultimately result in a different clinical outcome.

## Data Availability

The original contributions presented in the study are included in the article/Supplementary material, further inquiries can be directed to the corresponding author.
