# Supplementary material for: Multiple brain abscesses caused by Nocardia asiatica co-infection with Torque teno virus in an “immunocompetent” patient: a rare case report and literature review
Source: Front Med (Lausanne). 2025 Nov 12;12:1661345. doi: 10.3389/fmed.2025.1661345 (PMC12646876; doi:10.3389/fmed.2025.1661345)
Supplement: Supplementary file 1 [file Data_Sheet_1.pdf]

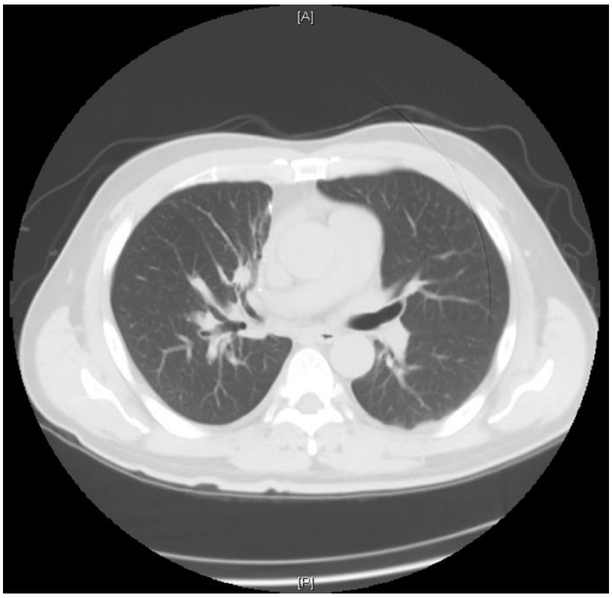

Figure S1 Contrast-enhanced CT shows a linear hyperdensity lesion in the right lung post-lobectomy, with associated right hilar enlargement and poorly defined borders.

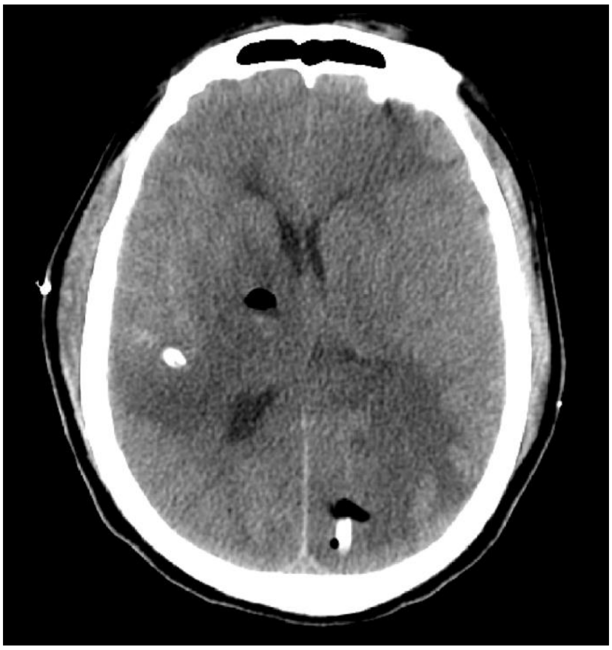

Figure S2 Head CT reveals multiple hypodense areas in the brain, along with tubular hyperdense structures in the left parietal and right temporal lobes post stereotactic drainage.
